# On the Determination of Elastic Properties of Single-Walled Nitride Nanotubes Using Numerical Simulation

**DOI:** 10.3390/ma17102444

**Published:** 2024-05-18

**Authors:** Nataliya A. Sakharova, André F. G. Pereira, Jorge M. Antunes, Bruno M. Chaparro, Tomás G. Parreira, José V. Fernandes

**Affiliations:** 1 Centre for Mechanical Engineering, Materials and Processes (CEMMPRE)—Advanced Production and Intelligent Systems, Associated Laboratory (ARISE), Department of Mechanical Engineering, University of Coimbra, Rua Luís Reis Santos, Pinhal de Marrocos, 3030-788 Coimbra, Portugal; andre.pereira@uc.pt (A.F.G.P.); jorge.antunes@dem.uc.pt (J.M.A.); tomas.parreira@dem.uc.pt (T.G.P.); valdemar.fernandes@dem.uc.pt (J.V.F.); 2 Abrantes High School of Technology, Polytechnic Institute of Tomar, Quinta do Contador, Estrada da Serra, 2300-313 Tomar, Portugal; bruno.chaparro@ipt.pt

**Keywords:** 13th group element, nitride nanotubes, rigidity, elastic moduli, modelling, numerical simulation

## Abstract

In recent years, tubular nanostructures have been related to immense advances in various fields of science and technology. Considerable research efforts have been centred on the theoretical prediction and manufacturing of non-carbon nanotubes (NTs), which meet modern requirements for the development of novel devices and systems. In this context, diatomic inorganic nanotubes formed by atoms of elements from the 13th group of the periodic table (B, Al, Ga, In, Tl) and nitrogen (N) have received much research attention. In this study, the elastic properties of single-walled boron nitride, aluminium nitride, gallium nitride, indium nitride, and thallium nitride nanotubes were assessed numerically using the nanoscale continuum modelling approach (also called molecular structural mechanics). The elastic properties (rigidities, surface Young’s and shear moduli, and Poisson’s ratio) of nitride nanotubes are discussed with respect to the bond length of the corresponding diatomic hexagonal lattice. The results obtained contribute to a better understanding of the mechanical response of nitride compound-based nanotubes, covering a broad range, from the well-studied boron nitride NTs to the hypothetical thallium nitride NTs.

## 1. Introduction

Compounds of atoms of the 13th group of the periodic table, such as boron (B), aluminium (Al), gallium (Ga), indium (In), and thallium (Tl), with nitrogen (N), representative of the 15th group, are emerging materials that are attractive for the electronic engineering and light industries. The ability of 13th group-nitrides to form a hexagonal graphene-like lattice [1] allows for the expansion of the area of their upcoming applications and brings to light new perspectives in the miniaturisation and designing of functional devices [2,3,4]. Hexagonal boron nitride (h-BN) is a high-strength electric insulator, comparable with graphene, having excellent thermal and chemical stability as well as transparency for visible light [5,6,7]. Such characteristics make h-BN suitable for diverse applications as a dielectric in graphene electronics, components for photovoltaic devices, sensors, and bio-detectors. Hexagonal aluminium nitride (h-AlN), gallium nitride (h-GaN), and indium nitride (h-InN), which also exhibit good thermal and chemical stability, are wide-gap semiconductors and are able of emitting light in colours green, blue, and UV bands [8]. As a result, these hexagonal metal nitrides (AlN, GaN, and InN) are in the focus of research attention due to their promising applications in electronics and optoelectronics as solid-state light-emitting devices (LEDs) and high-speed field-effect transistors (FETs) [1,4,9]. Hexagonal thallium nitride (h-TlN) has a small or even negative energy band gap [10] pointing out to its semi-metallic nature. This makes h-TlN an appropriate candidate for infrared optical devices [3,11].

One-dimensional (1D) tubular nanostructures, i.e., nanotubes (NTs), composed of hexagonal BN, AlN, GaN, InN, and TlN monolayers, are expected to have enhanced properties when compared with their bulk counterparts, envisioning new perspectives in the development of nanoscale electronic and light devices but not being limited to them. For example, boron nitride nanotubes have the potential to be used in the smallest co-axial cable, a possibility that was unlocked when a carbon nanotube was grown inside of it [12]. The high surface-to-volume ratio of NTs suggests their forthcoming applications for gas absorption and as chemical sensors. The possibility of tuning electronic, thermoelectric, optical, and chemical properties of two-dimensional (2D) 13th group-nitride nanostructures through the introduction of deformation [1,4,13,14,15,16,17] points to a promising use of their 1D allotropes in the field of strain engineering. In view of the abovementioned perspectives, viable applications of boron nitride NTs as biosensors [18] and aluminium nitride NTs as gas adsorbents [19] and for drug delivery [20], as well as the suitability of gallium nitride NTs for nanoelectromechanical systems (NEMSs) [21], were considered.

Most nanotubes based on the 13th group-nitride compounds are by now predicted and synthesised. After the theoretical prediction of the boron nitride nanotube (BNNT) in 1994 [22] and its synthetisation in 1995 by Chowdhury and Adhikari [18], who used arc discharge processing, BN nanotubes with a honeycomb atomic arrangement were successfully manufactured using chemical vapour deposition (CVD) [23,24], ball milling [25], laser ablation [26], and thermal plasma jet [27] processing. Unlike BNNTs, progress in synthesising AlN, GaN, and InN nanotubes is to some extent limited. In 2003, Zhang and Zhang [28] performed a theoretical study on the stability of the geometrical structure of aluminium nitride nanotubes (AlNNTs) and provided perspectives for their synthesis. It was concluded that the Al and N atoms form a hexagonal graphene-like arrangement, carrying out sp^2^ hybridisation [28]. Wu et al. [29] synthesised AlNNTs through a nitriding reaction in the same year. The proposed growth method made it possible to obtain faceted AlNNTs with a length of a few micrometres and a hexagonal cross-section. Balasubramanian et al. [30] grew AlNNTs using gas-phase condensation using a solid–vapour equilibrium. The atomic structure of the resulting AlNNTs consisted of hexagonal rings of Al and N atoms, which adopt sp^2^ hybridisation. Yin et al. [31] produced C–AlN–C coaxial composite NTs in mass quantity by resorting to a chemical substitution reaction in a controllable two-step process with the use of multi-walled carbon nanotubes (MWCNTs) as a template. The AlNNTs obtained were straight, several micrometres long, and had a faceted single-crystalline structure. Stan et al. [32] synthesised faceted AlNNTs with a triangular cross-section through an epitaxial casting process that consisted of the depositing of aluminium nitride onto GaN nanowires, which were subsequently removed by annealing in a hydrogen atmosphere so that the AlN tubes remained hollow. Finally, one-micrometre-long AlNNTs with hexagonal wurtzite structure were synthesised by Fan et al. [33], who used a thermal process to bend and roll up the AlN monolayer for this purpose. With respect to gallium nitride nanotubes (GaNNTs), their structural stability and prospects of synthesis were first theoretically investigated in 1999 by Lee et al. [34] based on the density functional theory (DFT) calculations. Then, in 2003, Goldberger et al. [35] prepared single-crystalline GaNNTs with hexagonal cross-sections, using an epitaxial casting method and ZnO nanowires as templates. Yin et al. [36] synthesised amorphous GaNNTs of a few micrometres in length using an In-assisted thermal evaporation process. Hu et al. [37] accomplished mass-quantity growth of straight crystalline GaNNTs with lengths of up to 80 μm using a two-stage process based on the controllable conversion of amorphous gallium oxide NTs. Hung et al. [38] synthesised uniform arrays of free-standing hexagonal GaNNTs on a GaN template using inductively coupled plasma etching. Liu et al. [39] manufactured single-crystalline hexagonal wurtzite-type GaNNTs based on a controllable chemical thermal evaporation process. Jung et al. [40] fabricated long crystalline GaNNTs aided by the metal organic chemical vapour deposition (MOCVD) technique. Concerning indium nitride nanotubes (InNNTs), in 2004, Yin et al. [41] for the first time synthesised straight, high-purity, crystalline InNNTs of several micrometres length in a large amount through a carbonitridation reaction in a vapour–solid (VS) route, with MWCNTs being the carbon source to carry out the chemical reaction. Soon after, Sardar et al. [42] produced almost defect-free single-crystalline InNNTs by employing the low-temperature chemical reaction of indium acetate with hexamethyldisilazane (HMDS). At about the same time, the theoretical prediction of InNNTs with stable, honeycomb graphene-like structures was accomplished by Qian et al. [43], who used DFT calculations to this end.

Among the 13th group-nitrides, TlN is the least studied, and nanostructures based on this compound have not been synthesised, possibly due to the high toxicity of thallium [2]. Despite the existence of several theoretical works dedicated to the structural stability of 2D TlN nanostructures with planar honeycomb atomic arrangement [44,45,46] and the evaluation of their electronic [3,4] and mechanical [1,46] properties, thallium nitride nanotubes (TlNNTs) have not yet been predicted. The structural similarity of h-TlN with other representatives of the 13th group-nitrides suggests that TlNNTs will possibly be modelled and synthesised in the future. The inclusion of these hypothetical nanotubes in the current study envisages expanding the range of the potential applications of 13th group-nitride NTs and meets the requirements for the search of new materials for innovative nanodevices.

The mechanical stability of nanotubes and the knowledge on their mechanical behaviour are crucial for current and forthcoming applications involving NTs, as well as for the design of materials and instruments. It is worth noting that strain engineering is efficient to customise the functional properties of nanomaterials. From this point of view, the evaluation of the mechanical properties of the 13th group-nitride NTs garners the utmost importance.

The study of the mechanical behaviour of non-carbon nanotubes (N-CNTs), whose representatives are those based on nitride compounds, has been performed mostly theoretically, aided by analytical and numerical procedures, because experimental techniques for nanomaterials characterisation are expensive and highly resource-consuming. As reported by Antunes et al. [47], the mechanical behaviour of N-CNTs can be characterised using three categories of theoretical methods, viz.: the atomistic approach, embracing ab initio and molecular dynamics (MD); the continuum mechanics (CM) approach; and the nanoscale continuum modelling (NCM) or molecular structural mechanics (MSM) approach. Amongst 13th group-nitride nanotubes, BNNTs have received the most research attention to date [47,48].

Referring to the atomistic approach, Kochaev [49] evaluated the product of the Young’s modulus and nanotube wall thickness, i.e., surface Young’s modulus and Poisson’s ratio of BNNTs, AlNNTs, and GaNNTs, making use of an ab initio simulation. Hao et al. [50] studied the mechanical behaviour of the AlNNTs and evaluated their Young’s modulus employing ab initio calculations with a linear combination of atomic orbitals (LCAO). Fabris et al. [51] used the same method to calculate the Young’s modulus of GaNNTs. Current studies, involving MD, rely on potential functions (analytical or empirical) to describe the atoms interactions in the hexagonal diatomic lattice. The second-generation reactive empirical bond order (REBO) potential was used by Kumar et al. [52] in their MD simulation study to estimate the elastic moduli and Poisson’s ratio of BNNTs, AlNNTs, and GaNNTs. Jeng et al. [53] employed MD simulation with the Tersoff many-body potential to describe the mechanical response of GaNNTs under tension and calculated their Young’s modulus. Xiong and Tian [54] studied the torsional properties of BNNTs, making use of MD simulation with the Tersoff potential and calculated the BNNTs’ shear modulus. Tao et al. [55] used MD with the Tersoff–Brenner (TB) potential to calculate the Young’s modulus of BNNTs. The Stilliger–Weber (S-W) potential was employed to describe the interactions between Ga and N atoms by Xu et al. [56] in their MD simulation study for evaluating the Young’s modulus of single-crystalline GaNNTs. Santosh et al. [57], with the aim of calculating the BNNTs Young’s and shear moduli, implemented the force constant approach to describe the B - N interactions under MD simulation. Le [58], based on MD simulations coupled with a dimensional analysis, derived analytical expressions for the Young’s modulus of the BNNTs.

With respect to the CM approach, which models the nanotube as a continuum structure, Oh [59] employed a continuum lattice (CL) analytical thermodynamic method in combination with the TB potential to calculate the elastic properties of BNNTs.

In the NCM/MSM approach, the interatomic bonds in the diatomic lattice are modelled as elastic elements (e.g., beams or springs), thus being based on the connection between NTs molecular structure and solid mechanics. In two of their works, Sakharova et al. [48,60] used the beam element to represent interatomic bonding within the NCM/MSM approach framework to determine the Young’s and shear moduli and the Poisson’s ratio of BNNTs [48] and InNNTs [60]. The latter, to the best of our knowledge, is the only study devoted to the elastic properties of InNNTs. Employing the NCM/MSM approach combined with the Euler beam model, Yan et al. [61] evaluated the elastic moduli of BNNTs, resorting to longitudinal and torsional free vibrations of nanotubes. Genoese et al. [62] calculated the surface Young’s and shear moduli of BNNTs based on a link between the “stick-and-spring” (NCM/MSM) and the Donnell thin-shell continuum models (CMs). The “stick-and-spring” model for deriving analytical solutions for the surface Young’s modulus and Poisson’s ratio of BNNTs, AlNNTs, and GaNNTs was also used by Jiang and Guo [63].

There are also some works dealing with the experimental evaluation of the elastic properties of BNNTs, AlNNTs and GaNNTs. Arenal et al. [64] evaluated the Young’s modulus of single-walled BNNTs from the results of in situ uniaxial compression tests carried out by high-resolution transmission-electron microscopy (HRTEM) and atomic force microscopy (AFM). Tanur et al. [65] evaluated the Young’s modulus of multi-walled boron nitride nanotubes (MWBNNTs) using a three-point bending technique in AFM. Zhou et al. [66] employed a high-order resonance technique within HRTEM to this end. Chen et al. [67] calculated the Young’s modulus of the MWBNNT from the directly measured critical compressive force, using transmission electron microscopy (TEM). Stan et al. [32] carried out experimental measurements of the Young’s modulus of faceted AlNNTs with a triangular cross-section by contact resonance atomic force microscopy (CR-AFM). Hung et al. [38] calculated the Young’s modulus and Poisson’s ratio of single-walled GaNNTs based on the nanoindentation technique.

It can be concluded that with the exception of BNNTs, studies on the mechanical characterisation of NTs based on other nitrides of the 13th group are limited (AlNNTs, GaNNTs), infrequent (InNNTs), or absent (TlNNTs). A lack of systematised investigation of the mechanical response of the nanotubes formed by nitride compounds is also noticeable. The current study aims to fill this gap.

The objective of this work is to perform a systematic comparative study on the evaluation of the surface elastic (Young’s and shear) moduli and Poisson’s ratio of single-walled nanotubes composed of boron nitride, aluminium nitride, gallium nitride, indium nitride, and thallium nitride (SWBNNTs, SWAlNNTs, SWGaNNTs, SWInNNTs, and SWTlNNTs) in a wide range of chiral indices and diameters greater than 1.25 nm. For this, a three-dimensional finite element (FE) model was built within the scope of the NCM/MSM approach to assess three rigidities (tensile, bending, and torsional) and calculate the surface Young’s and shear moduli and Poisson’s ratio of the 13th group-nitride nanotubes. In view of the lack of information on the value of nanotube wall thickness for nitride NTs, except in the case of SWBNNTs, the surface elastic moduli were chosen for this analysis. The present work aims to improve the understanding of the mechanical response of the nitride nanotubes, which groups materials with insulator, semiconductor, and semi-metallic properties. The results allow us to unlock new perspectives for the use nitride nanotubes in innovative devices and their accurate design and robust performance.

## 2. Materials and Methods

### 2.1. Atomic Structure of 13th Group Element—Nitride Nanotubes

**Figure 1:**
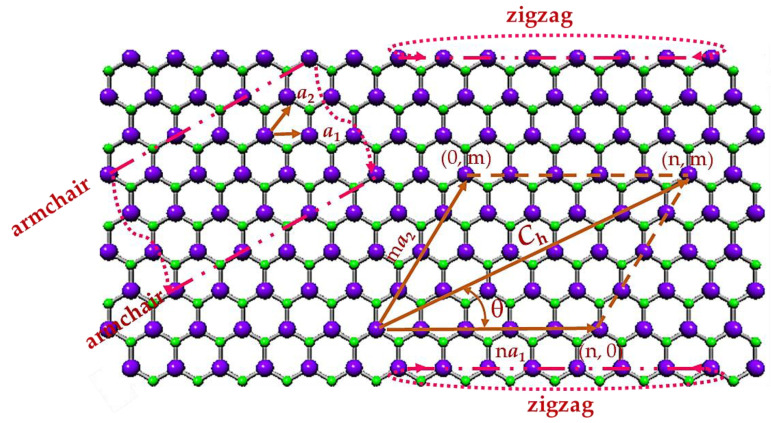
GaN hexagonal nanosheet with designations of the chiral vector, **C_h_**, chiral indices, n and m, and chiral angle, θ, and the schematic to roll up zigzag and armchair NTs geometries. Ga atoms are depicted in purple; N atoms are depicted in green.

Boron nitride, aluminium nitride, gallium nitride, indium nitride, and thallium nitride sheets have a hexagonal lattice, where the atom that is part of the 13th group of the periodic table (now designated A13), such as boron (B), aluminium (Al), gallium (Ga), indium (In), or thallium (Tl), form with nitrogen (N) into a honeycomb structure. For all nitride compounds under study, the hexagonal lattice has planar geometry [1,68], as shown for the case of the GaN nanosheet in Figure 1. The honeycomb atomic arrangement is defined by the chiral vector, **C_h_**, and the chiral angle, θ, expressed as follows, respectively:

(1) $$C_{h}=na_{1}+ma_{2},$$

(2) $$\theta =\sin^{-1}\frac{\sqrt{3}}{2}\frac{m}{\sqrt{n^{2}+\mathrm{nm}+m^{2}}},$$

where n and m are the chiral indices, both having integers values; $a_{1}$ and $a_{2}$ are the unit vectors of the diatomic hexagonal lattice. The length of the unit vector a is calculated by $a=\sqrt{3}a_{A13-N}$, where $a_{A13-N}$ is the equilibrium bond length. As can be seen in Table 1, where the bond lengths of nitride NTs available in the literature are presented, there is no conformity about the $a_{A13-N}$ values.

Single-walled nitride NTs are cylinders, which are formed by rolling up the respective A13-N nanosheet with a honeycomb atomic arrangement, varying the chiral angle, θ, in the range of 0° ≤ θ ≤ 30°. The diameter of resulting nanotube, $D_{n}$, is given by:

(3) $$D_{n}=\frac{a_{A13-N}\sqrt{3\left(n^{2}+\mathrm{nm}+m^{2}\right)}}{\pi },$$

where n and m are the chiral indices and $a_{A13-N}$ is the equilibrium bond length of the diatomic nanostructure based on the nitride compounds under study.

Three NT symmetry groups are defined based on the θ value, such as: zigzag (n, 0) NTs with θ = 0° (m = 0); chiral (n, m) NTs with 0° < θ < 30° (n ≠ m ≠ 0); and armchair (n, n) NTs with θ = 30° (n = m). The configurations, limiting the range of θ, viz. (n, 0) zigzag and (n, n) armchair (see, Figure 1), are designated as non-chiral nanotubes.

**Figure 2:**
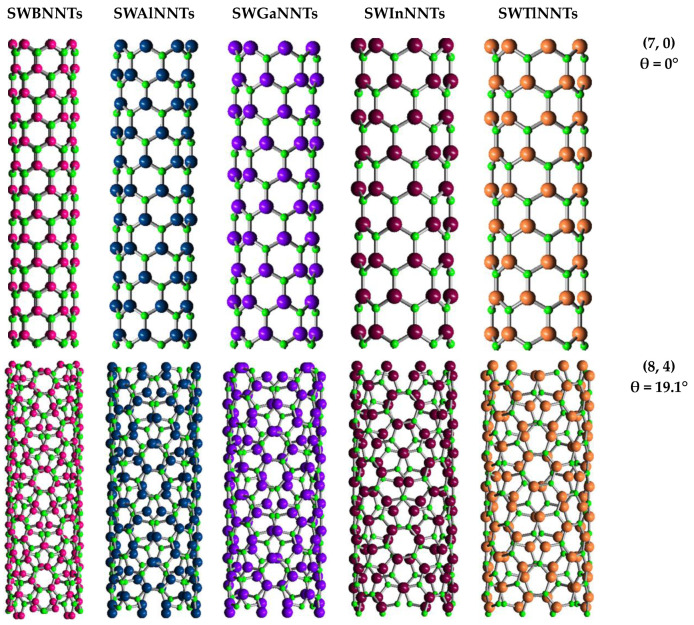

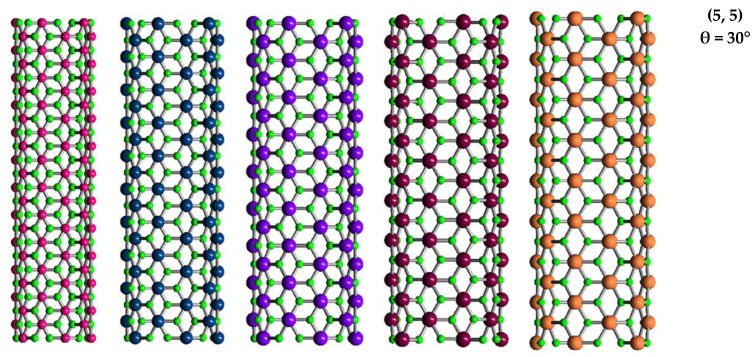
Structures of (7, 0) zigzag, (8, 4) chiral and (5, 5) armchair of SWBNNTs, SWAlNNTs, SWGaNNTs, SWInNNTs, and SWTlNNTs, acquired using Nanotube Modeler© software (version 1.8.0, ©JCrystalSoft, http://www.jcrystal.com, 1 March 2024). N atoms are depicted in green, and B atoms are depicted in bright pink, Al atoms are in blue, Ga atoms are in purple, In atoms are in dark red, and Tl atoms are in pale orange.

Non-chiral (zigzag and armchair) and chiral SWBNNTs, SWAlNNTs, SWGaNNTs, SWInNNTs, and SWTlNNTs, with the same chiral indices (n, m) for each symmetry group, are represented schematically in Figure 2.

### 2.2. Geometrical Characteristics and Finite Element Modeling of the Elastic Behaviour of SWBNNTs, SWAlNNTs, SWGaNNTs, SWInNNTs, and SWTlNNTs

The geometric characteristics of SWBNNTs, SWAlNNTs, SWGaNNTs, SWInNNTs, and SWTlNNTs of three main configurations, armchair (θ = 30°), zigzag (θ = 0°), and chiral (θ = 19.1° family, which is consistent with the biggest number of NTs), used in the finite element analysis (FEA), are shown in Table 2. The NT chiral indices were chosen to obtain structures with similar diameters. To guarantee the mechanical response of the NTs regardless of nanotube length, the length of the NTs was nearly 30 times bigger than the diameter of the NTs [48].

**Figure 3:**
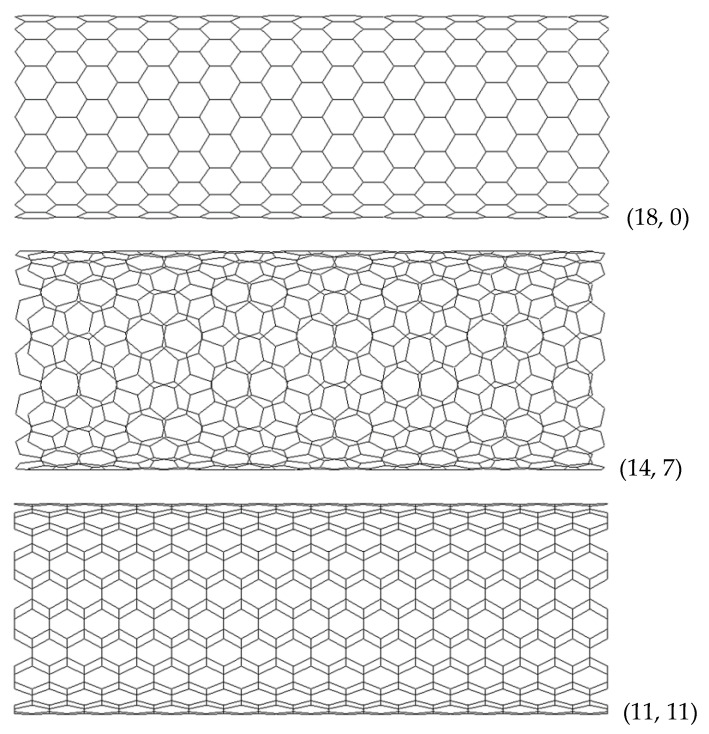
FE meshes of zigzag (18, 0), chiral (14, 7), and armchair (11, 11) InN nanotubes.

The FE meshes of SWBNNTs, SWAlNNTs, SWGaNNTs, SWInNNTs, and SWTlNNTs used in the FE analysis were built utilizing the Nanotube Modeler© software. The program database files acquired from this software were converted to the format supported by the ABAQUS^®^ code (Abaqus 2020, Dassault Systèmes®). The in-house program InterfaceNanotubes.NM [48] was used for this purpose. FE meshes for zigzag, chiral, and armchair InNNTs are exemplified in Figure 3.

The interatomic bonds, A13-N of the hexagonal NTs lattice, were modelled as equivalent beam elements within the framework of the NCM/MSM method, which makes use of the linking between the nanotube molecular structure and the equivalent continuum structure. The latter is composed of beam elements and is characterised by its tensile E_b_A_b_, bending E_b_I_b_, and torsional G_b_J_b_ rigidities, which are related to the bond stretching k_r_, bond bending k_θ_, and torsional resistance k_τ_ force field constants, representing the corresponding molecular structure through the following expressions [75]:

(4) $$E_{b}A_{b}=lk_{r},\;E_{b}I_{b}=lk_{\theta },\;G_{b}J_{b}=lk_{\tau }$$

where $A_{b}=\pi d^{2}/4$ is the cross-section area, $I_{b}=\pi d^{4}/64$ is the moment of inertia, and $J_{b}=\pi d^{4}/32$ is the polar moment of inertia of a circular cross-section beam with diameter d and being *l* the beam length, equivalent to the bond length, $a_{A13-N}$.

Equation (4) allow for the computation of the numerical simulation input parameters utilising the $k_{r}$, $k_{\theta }$, and $k_{\tau }$ force field constants. Unlike BNNTs, for which several values of the bond stretching k_r_ and bond bending k_θ_, force constants are available in the literature [48], for the other 13th group elements-nitride NTs, these data are scarce or non-existent. For this reason, in the present study, the $k_{r}$ and $k_{\theta }$ force field constants were calculated resorting to the method that uses analytical molecular mechanics (MM) expressions for the surface Young’s modulus, $E_{s}$, and Poisson’s ratio, ν. The values of $E_{s}$ and ν, in turn, originate from DFT calculations or can be obtained experimentally. Thus, the bond stretching and bond bending force constants are derived by resolving the following system of equations [76]:

(5) $$\left\{\begin{array}{c} E_{s}=\frac{4\sqrt{3}k_{r}k_{\theta }}{k_{r}\frac{a_{A13-N}^{2}}{2}+9k_{\theta }}\\ \nu =\frac{k_{r}a_{A13-N}^{2}-6k_{\theta }}{k_{r}a_{A13-N}^{2}+18k_{\theta }}. \end{array}\right.$$

As a result, the $k_{r}$ and $k_{\theta }$ force field constants are assessed as follows:

(6) $$k_{r}=\frac{3E_{s}}{\sqrt{3}\left(1-\nu \right)},$$

(7) $$k_{\theta }=\frac{E_{s}a_{A13-N}^{2}}{2\sqrt{3}\left(1+3\nu \right)}.$$

The parameters $a_{A13-N}$, $E_{s}$, and ν, necessary for calculating the bond stretching k_r_ and bending k_θ_ force constants (Equation (6) and (7)), together with the calculated $k_{r}$ and $k_{\theta }$ values, are presented in Table 3.

With respect to the torsion resistance force constant, $k_{\tau }$, the value calculated by Ansari et al. [77], based on the relationship of the $k_{\tau }$ constant with the bending rigidity of the BN nanosheet, was adopted for SWBNNTs. For the remaining nitride NTs under study, the $k_{\tau }$ was acquired using the DREIDING force field [78], where the torsional behaviour is only defined by the hybridisation of the diatomic nanostructure atoms. The values used for $k_{\tau }$ are shown in Table 3.

Finally, based on Equation (4) and the values of $k_{r}$, $k_{\theta }$, and $k_{\tau }$ from Table 3, and taking into account the equality of the bond and beam lengths, $a_{A13-N}$ = *l*, it is possible to calculate the input values for the numerical simulation (geometrical and elastic properties of the beams) according to Table 4, together with their respective formulation.

### 2.3. Elastic Properties of SWBNNTs, SWAlNNTs, SWGaNNTs, SWInNNTs, and SWTlNNTs

**Figure 4:**
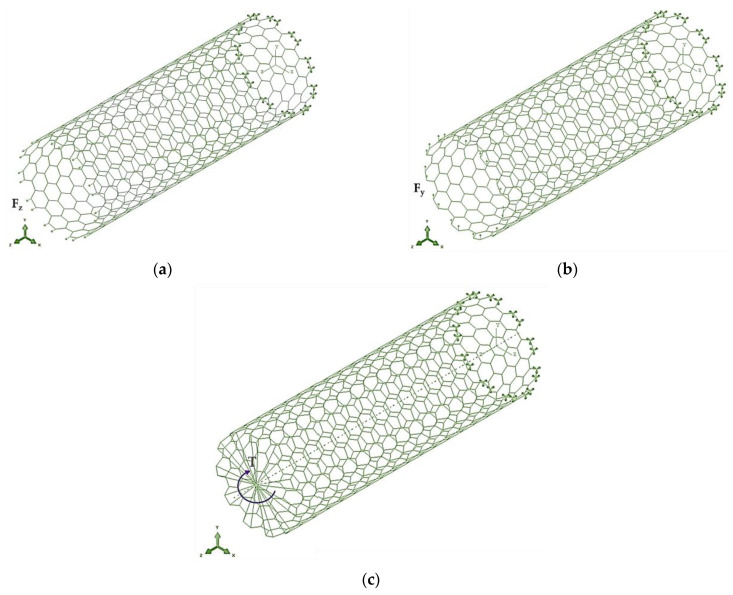
Boundary and loading conditions applied in tests of (**a**) tension, (**b**) bending, and (**c**) torsion of armchair SWInNNTs.

Firstly, the tensile EA, bending EI, and torsional GJ rigidities of the 13th group-nitride nanotubes were obtained from the results of the FE analysis, carrying out tensile, bending, and torsion tests using the ABAQUS^®^ FE code (see Figure 4). To carry out each abovementioned test, the axial force, $F_{z}$, the transverse force, $F_{y}$, and the torsional moment, T, were applied to one end of the NT. The boundary conditions applied at the opposed NT end restricted all degrees of freedom of the nodes involved. In the torsion test, an additional boundary condition was imposed, which consists of preventing the edge nodes from moving in the radial direction, as shown in Figure 4c. Consequently, the tensile, bending, and torsion tests made it possible to acquire the axial displacement $u_{z}$, the transverse displacement $u_{y}$, and the twist angle φ directly from the FEA. These results are used to calculate the tensile EA, bending EI, and torsional GJ, rigidities of the nitride NTs with length $L_{n}$ as follows:

(8) $$\mathrm{EA}=\frac{F_{z}L_{n}}{u_{z}},$$

(9) $$\mathrm{EI}=\frac{F_{y}L_{n}^{3}}{{3u}_{y}},$$

(10) $$\mathrm{GJ}=\frac{TL_{n}}{\varphi }.$$

The tensile, EA, and bending, EI, rigidities from Equation (8) and (9), respectively, are required to assess the Young’s modulus, E, of the nitride NTs as follows [79]:

(11) $$E=\frac{\mathrm{EA}}{\pi t_{n}\sqrt{8\left(\frac{\mathrm{EI}}{\mathrm{EA}}\right)–t_{n}^{2}}},$$

where $t_{n}$ is the nanotube wall thickness. The knowledge of the valid value of $t_{n}$ is not available for most of the 13th group element-nitride NTs, except for BNNTs.

To calculate the shear modulus G, the torsional rigidity GJ, obtained by Equation (10), is needed in addition to the EA and EI rigidities. The evaluation of the Poisson’s ratio ν is based on the bending EI and torsional GJ rigidities, and ν is independent of the value of $t_{n}$. The following expressions are used for the assessment of G and ν [80]:

(12) $$G=\frac{\mathrm{GJ}}{2\pi t_{n}\left(\frac{\mathrm{EI}}{\mathrm{EA}}\right)\sqrt{8\left(\frac{\mathrm{EI}}{\mathrm{EA}}\right)–t_{n}^{2}}},$$

(13) $$\nu =\frac{E}{2G}\;-\;1=\frac{\mathrm{EI}}{\mathrm{GJ}}\;-\;1.$$

Assuming the uncertainty of the values of the NT wall thickness, the surface elastic moduli, Young’s ($E_{S}=Et_{n}$) and shear ($G_{S}=Gt_{n}$) moduli, were evaluated in the present study. In fact, the $E_{s}$ and $G_{s}$ elastic moduli are more reliable to describe the mechanical response of the nitride NTs as they do not depend on the wall thickness. Considering that $t_{n}^{2}\;\ll \;8\left(\frac{\mathrm{EI}}{\mathrm{EA}}\right)$ and that the term $t_{n}^{2}$ in Equations (11) and (12) can be neglected, the surface Young’s, $E_{S}$, and shear, $G_{S}$, moduli are determined, respectively, as follows:

(14) $$E_{S}=Et_{n}=\frac{\mathrm{EA}}{\pi \sqrt{8\left(\frac{\mathrm{EI}}{\mathrm{EA}}\right)}},$$

(15) $$G_{S}=Gt_{n}=\frac{\mathrm{GJ}}{2\pi \left(\frac{\mathrm{EI}}{\mathrm{EA}}\right)\sqrt{8\left(\frac{\mathrm{EI}}{\mathrm{EA}}\right)}}.$$

## 3. Results and Discussion: Elastic Properties of SWBNNTs, SWAlNNTs, SWGaNNTs, SWInNNTs, and SWTlNNTs

### 3.1. Rigidities

**Figure 5:**
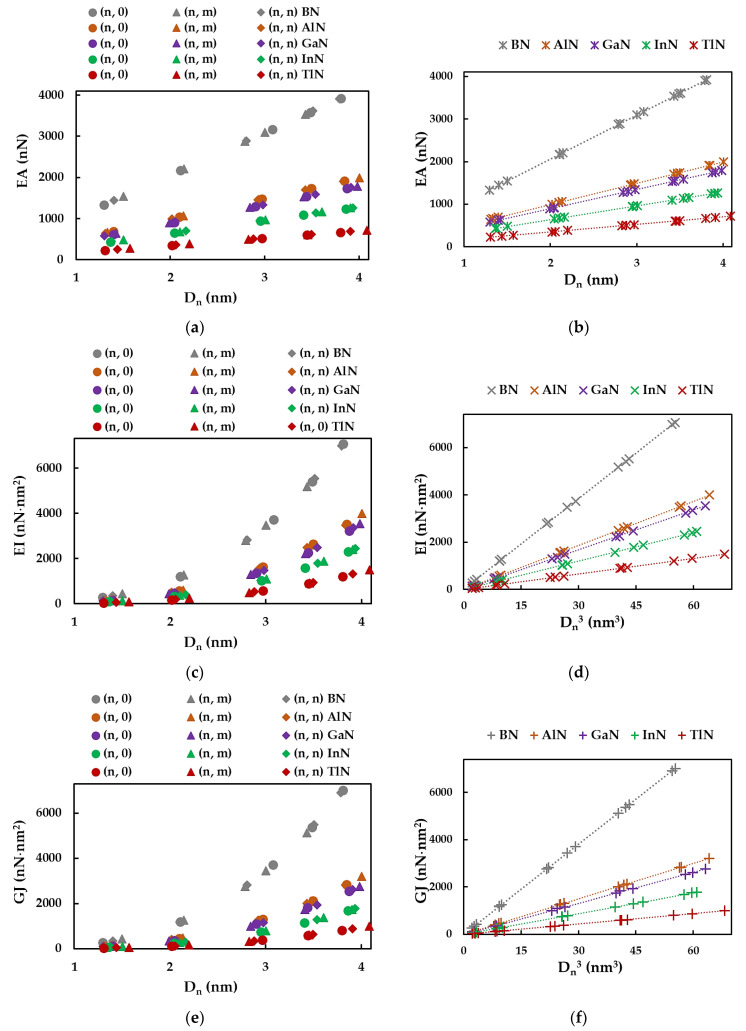
Evolutions of (**a**,**b**) tensile, EA, (**c**) bending, EI, and (**e**) torsional, GJ, rigidities as a function of the NT diameter, $D_{n}$; (**d**) bending, EI, and (**f**) torsional, GJ, rigidities as a function of $D_{n}^{3}$ for the SWBNNTs, SWAlNNTs, SWGaNNTs, SWInNNTs, and SWTlNNTs in Table 2.

The tensile, bending, and torsional (EA, EI, and GJ) rigidities of the SWBNNTs, SWAlNNTs, SWGaNNTs, SWInNNTs, and SWTlNNTs, calculated by Equations (8)–(10) from the FEA results, are plotted as a function of the nanotube diameter $D_{n}$ in Figure 5a,c,e. For each of the three rigidities, the same trend is observed with the increase in the nanotube diameter regardless of the NTs symmetry group (zigzag, chiral, or armchair) and nanotube compound. It is worth mentioning that the EA, EI, and GJ rigidities decrease from the values obtained for SWBNNTs to those for SWTlNNTs. As previously established for the phosphide [81] and carbide [82,83] nanotubes, in the case of the 13th group element-nitride NTs, the tensile rigidity EA can be described by a linear function of $D_{n}$ (Figure 5a,b), while the bending EI and torsional GJ rigidities can be described by a linear function of $D_{n}^{3}$ (Figure 5c–f).

Similar to what was established in the authors’ earlier studies for the phosphide [77] and carbide [78,79] nanotubes, the slope of the straight lines in Figure 5b,d,f can be determined as follows:

(16) $$\mathrm{EA}=\alpha_{A13-N}D_{n},$$

(17) $$\mathrm{EI}=\beta_{A13-N}{D_{n}}^{3},$$

(18) $$\mathrm{GJ}=\gamma_{A13-N}{D_{n}}^{3}.$$

In these equations, $\alpha_{A13-N}$, $\beta_{A13-N}$, and $\gamma_{A13-N}$ are fitting parameters, and $D_{n}$ is the diameter of the nitride NTs. The values of these parameters were determined as the slope of the dashed lines in the graphs of Figure 5b,d,f, with the R-squared values always being better than 0.9999, regardless of the rigidity and compound forming the nanotubes. The $\alpha_{A13-N}$, $\beta_{A13-N}$, and $\gamma_{A13-N}$ fitting parameters, together with the mean differences between the values of EA, EI, and GJ assessed by the analytical expressions (16)–(18) and those derived from FEA (Equations (8)–(10)), are shown in Table 5. It can be seen in this table that the mean difference does not exceed 0.58%. Therefore, Equations (16)–(18) result in accurate values of the three rigidities of SWBNNTs, SWAlNNTs, SWGaNNTs, SWInNNTs, and SWTlNNTs and can be used to evaluate the EA, EI, and GJ rigidities without the resource of numerical simulation.

**Figure 6:**
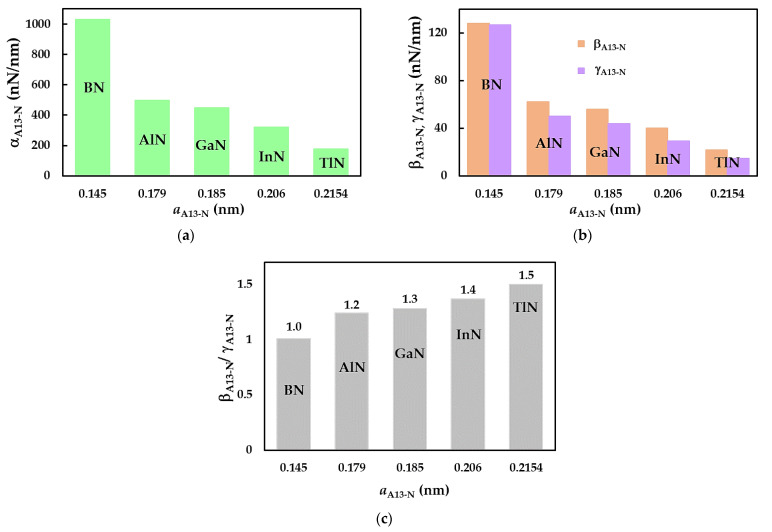
Fitting parameters as a function of the bond lengths, $a_{A13-N}$: (**a**)$\alpha_{A13-N}$; (**b**) $\beta_{A13-N}$ together with $\gamma_{A13-N}$; and (**c**) $\beta_{A13-N}$/$\gamma_{A13-N}$ ratio for SWBNNTs, SWAlNNTs, SWGaNNTs, SWInNNTs, and SWTlNNTs.

The fitting parameters $\alpha_{A13-N}$, $\beta_{A13-N}$, and $\gamma_{A13-N}$ in Table 5 allow for the quantification of the tensile, bending, and torsional rigidities, respectively, thereby describing the mechanical response of nanotubes under tension, bending, and torsion. To this end, the values of $\alpha_{A13-N}$ and $\beta_{A13-N}$, together with $\gamma_{A13-N}$, are presented in Figure 6a and 6b, respectively, for the bond lengths, $a_{A13-N}$, corresponding to the SWBNNTs, SWAlNNTs, SWGaNNTs, SWInNNTs, and SWTlNNTs. All three fitting parameters drop from SWBNNTs to SWAlNNTs; then, the $\alpha_{A13-N}$, $\beta_{A13-N}$, and $\gamma_{A13-N}$ values decrease gradually when moving to SWTlNNTs, i.e., as the $a_{A13-N}$ value increases (see Figure 6a,b). With respect to the relationship between bending EI and torsional GJ rigidities, which can be defined by the ratio between the respective fitting parameters $\beta_{A13-N}$ and $\gamma_{A13-N}$, the ratio is $\beta_{A13-N}$/$\gamma_{A13-N}$ ≈ 1 for the SWBNNTs (see Figure 6c). It suggests that in this case, the EI and GJ rigidities are practically identical. For the remaining cases, the ratio of $\beta_{A13-N}$/$\gamma_{A13-N}$ becomes nearly equal to 1.2 for the SWAlNNTs and continues increasing in increments of 0.1 up to $\beta_{A13-N}$/$\gamma_{A13-N}$ ≈ 1.5 for the SWTlNNTs. As seen in Figure 6c, increasing the bond length $a_{A13-N}$ leads to a decrease in torsional rigidity and, subsequently, to the more significant difference between the EI and GJ rigidities. It can be concluded that the SWInNNTs and SWTlNNTs with longer bond lengths, $a_{\mathrm{In}-N}$ = 0.206 nm and $a_{\mathrm{Tl}-N}$ = 0.215 nm, respectively, have weaker torsional properties when compared with those of the other 13th group atom-nitride NTs.

### 3.2. Surface Young’s Modulus

The surface Young’s modulus, $E_{S}$, of the SWBNNTs, SWAlNNTs, SWGaNNTs, SWInNNTs, and SWTlNNTs was assessed with the aid of Equation (14), which makes use of the numerical results of the tensile and bending tests. In addition, an analytical expression for $E_{S}$, independent of $D_{n}$, can be obtained. For this, the tensile EA and bending EI rigidities in Equation (14) are replaced by expression (16) and (17), leading to the following equation:

(19) $$E_{S}=\frac{\alpha_{A13-N}}{\pi \sqrt{8\left(\frac{\beta_{A13-N}}{\alpha_{A13-N}}\right)}}.$$

where $\alpha_{A13-N}$ and $\beta_{A13-N}$ are fitting parameters (see Table 5).

**Figure 7:**
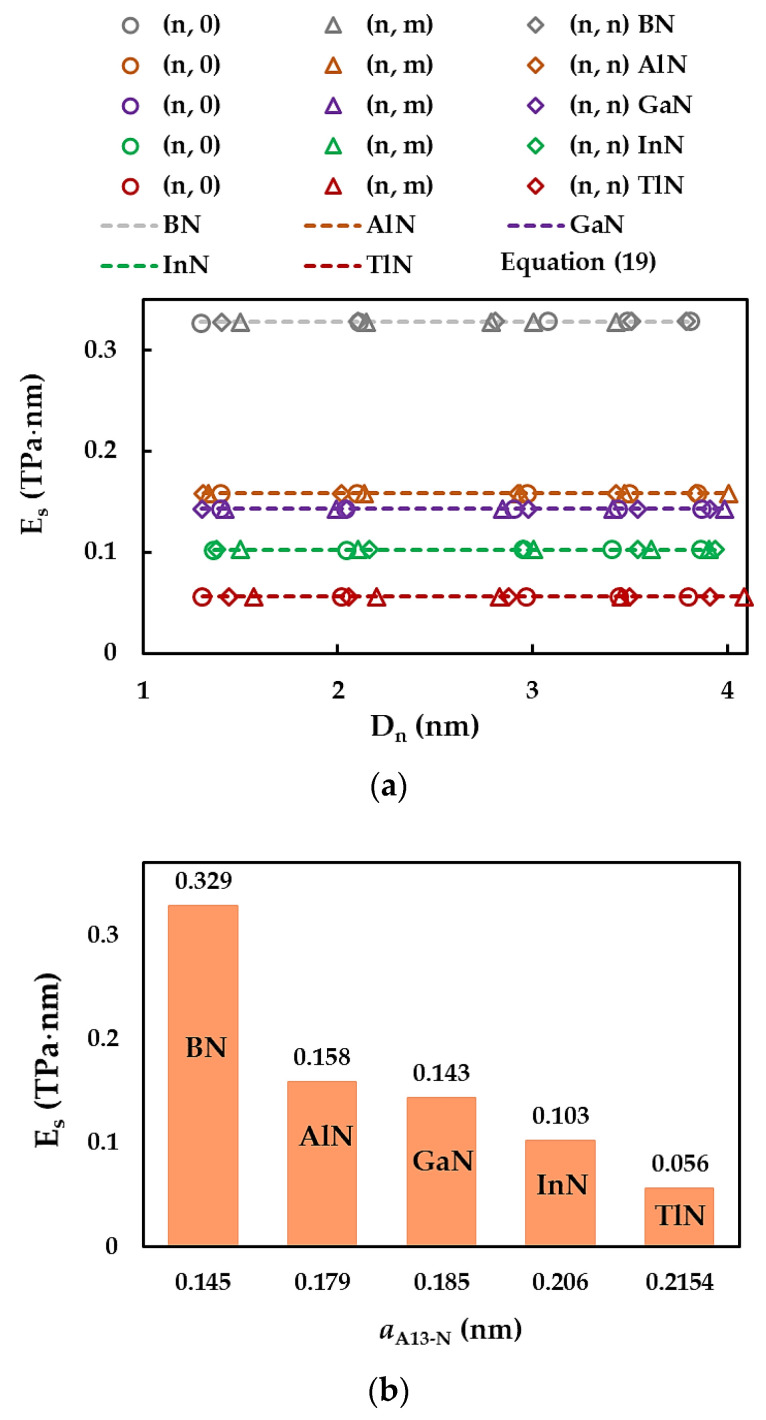
Evolutions of the surface Young’s modulus, $E_{S}$, for SWBNNTs, SWAlNNTs, SWGaNNTs, SWInNNTs, and SWTlNNTs as a function of the: (**a**) NT diameter, $D_{n}$; and (**b**) bond lengths, $a_{A13-N}$.

Figure 7a displays the evolutions of the surface Young’s modulus, $E_{S}$, assessed by Equation (14) with the NT diameter, $D_{n}$, for all single-walled nitride nanotubes studied. The results of $E_{S}$ calculated by Equation (19) are also plotted in Figure 7a by dashed lines. For the 13th group-nitride NTs, regardless of the chiral angle (zigzag, chiral, or armchair NTs) as well the compound (BN, AlN, GaN, InN, or TlN), the surface Young’s modulus is quasi-constant with increasing NTs diameter through the range of $D_{n}$ considered in the current work. It can be concluded from Figure 7a that Equation (19) permits an accurate evaluation of the surface Young’s modulus of nitride nanotubes. In fact, the mean differences between the values of $E_{S}$ evaluated with the aid of analytical Equation (19) and those determined from the FEA by Equation (14) are 0.18%, 0.08%, 0.10%, 0.12%, and 0.17% for the SWBNNTs, SWAlNNTs, SWGaNNTs, SWInNNTs, and SWTlNNTs, respectively. As a result, the elastic properties of the nitride NTs can be accurately evaluated without resorting to numerical simulation. To examine the influence of the first element (B, Al, Ga, In, Tl) of the nitride compound, which forms the nanotube on the surface Young’s modulus results, the $E_{S}$ values assessed by Equation (19) are plotted in Figure 7b considering the respective bond lengths $a_{A13-N}$.

The $E_{S}$ value decreases by almost half when moving from SWBNNTs to SWAlNNTs, and $E_{S}$ continues to decrease at a slower rate with increasing interatomic bond length, $a_{A13-N}$. The surface Young’s modulus of SWTlNNTs ($a_{\mathrm{Tl}-N}$ = 0.215 nm) is about six times lower when compared with that obtained for the SWBNNTs ($a_{B-N}$ = 0.145 nm). This decreasing tendency observed for the surface Young’s modulus with increasing bond length was reported by Jiang and Guo [68] as well for nitride and phosphide NTs and by Sakharova et al. [81] for phosphide NTs.

**Figure 8:**
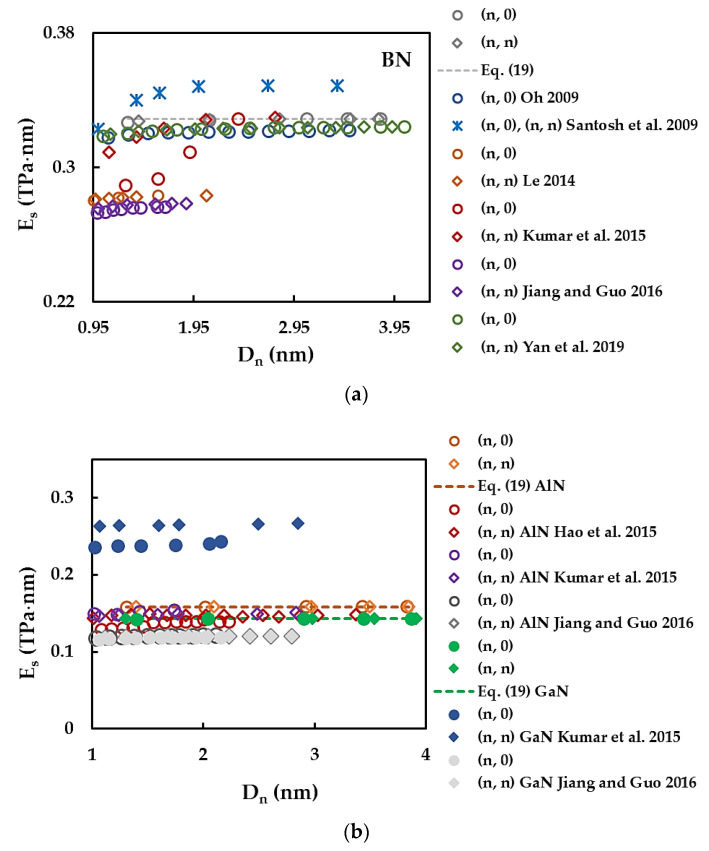
Comparison of the present evolutions of the surface Young’s modulus, $E_{S}$, with those available in the literature for: (**a**) SWBNNTs and (**b**) SWAlNNTs and SWGaNNTs as a function of the NT diameter, $D_{n}$ [50,52,57,58,59,61,63].

Figure 8 compares the surface Young’s modulus, $E_{S}$, values obtained for SWBNNTs (Figure 8a) and SWAlNNTs and SWGaNNTs (Figure 8b) with those available in the literature. A comprehensive comparison of the Young’s modulus results of SWBNNTs with the literature was carried out in a previous work by the authors [48], and therefore, only a few selected $E_{S}$ values were currently chosen. The $E_{S}$ results of Hao et al. [50], Kumar et al. [52], Santosh et al. [57], Oh [59], and Yan et al. [61] were assessed based on the Young’s modulus E by the equality $E_{S}=Et_{n}$ for NT wall thickness $t_{n}$ = 0.333 nm [61], 0.340 nm [57], 0.330 nm [52,59], and 0.410 nm [50].

A good agreement is observed when the present values of the SWBNNTs surface Young’s modulus are compared with those reported by Yan et al. [61] for non-chiral (zigzag and armchair) NTs (difference ≈ 1.30%), Oh [59] for zigzag NTs (difference ≈ 2.00%), and Kumar et al. [52] for armchair NTs with $D_{n}\;≳$ 1 nm (difference ≈ 0.36%) and zigzag NTs with $D_{n}\;≳$ 1.95 nm (difference ≈ 0.14%), as seen in Figure 8a. The $E_{S}$ values of the SWAlNNTs evaluated in the present study are about 4.6% and 6.9% lower than those obtained in the works by Kumar et al. [52] and Hao et al. [50], respectively (Figure 8b). With respect to SWGaNNTs, a scattering of the surface Young’s modulus results is noticeable (see Figure 8b). Whatever the case, whether they be SWAlNNTs or SWGaNNTs, more Young’s modulus results are needed to build a reliable benchmark to ascertain their elastic properties using theoretical methods.

The Young’s modulus experimental results available in the literature for boron nitride, aluminium nitride, and gallium nitride NTs are shown in Table 6.

It can be concluded that there is a reasonable agreement between the current values of the Young’s modulus and experimental values reported in the literature.

### 3.3. Surface Shear Modulus and Poisson’s Ratio

In this section, two elastic properties are discussed that require torsional rigidity in addition to the tensile and bending rigidities or just bending rigidity for their calculation, i.e., surface shear modulus and Poisson’s ratio, respectively.

The surface shear modulus, $G_{S}$, of the SWBNNTs, SWAlNNTs, SWGaNNTs, SWInNNTs, and SWTlNNTs was evaluated by Equation (15), which makes use of the results of the numerical tensile, bending, and torsional tests. Also, by substituting in Equation (15) the EA, EI, and GJ rigidities given by expressions (16)–(18), and with knowledge of the fitting parameters, $\alpha_{A13-N}$, $\beta_{A13-N}$, and $\gamma_{A13-N}$, from Table 5, $G_{S}$ can be assessed by the following analytical expression:

(20) $$G_{S}=\frac{\gamma_{A13-N}}{\pi \sqrt{32{\left(\frac{\beta_{A13-N}}{\alpha_{A13-N}}\right)}^{3}}},$$

which allows us to calculate $G_{S}$ without needing to know the NTs diameter, $D_{n}$.

**Figure 9:**
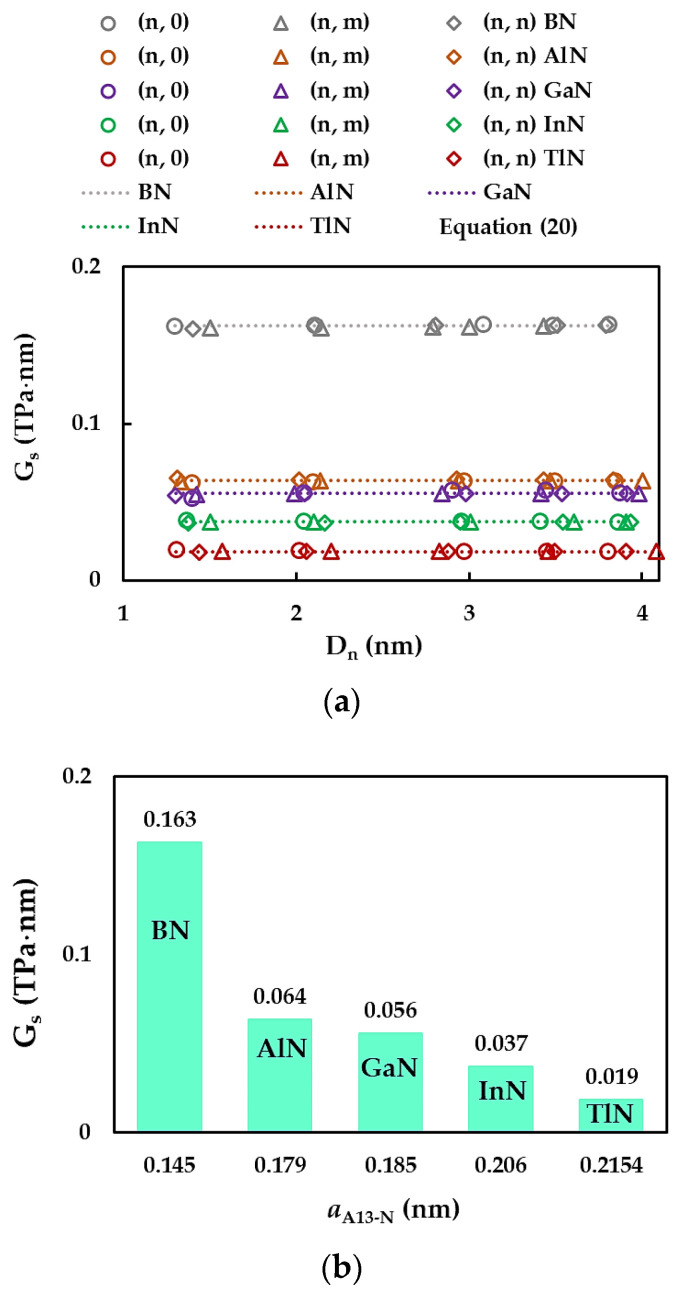
Evolutions of the surface shear modulus, $G_{S}$, for SWBNNTs, SWAlNNTs, SWGaNNTs, SWInNNTs, and SWTlNNTs as a function of the (**a**) NT diameter, $D_{n}$, and (**b**) bond length, $a_{A13-N}$.

The evolutions of the surface shear modulus, $G_{S}$, assessed by Equation (15) as a function of the NT diameter, $D_{n}$, together with the $G_{S}$ values evaluated using Equation (20), are plotted in Figure 9a for the cases of SWBNNTs, SWAlNNTs, SWGaNNTs, SWInNNTs, and SWTlNNTs. For nitride nanotubes, regardless of the NTs symmetry and the first element of the nitride compound forming the NT, the surface shear modulus is quasi-constant with increasing $D_{n}$. The mean differences between the $G_{S}$ values calculated analytically with the help of Equation (20) and those acquired from the FEA results by Equation (15) are 0.13%, 0.17%, 0.59%, 0.19%, and 0.26% (1.46%, 1.74%, 2.83%, 2.45%, and 1.97% for NTs with $D_{n}$ ≲ 1.5 nm), for the SWBNNTs, SWAlNNTs, SWGaNNTs, SWInNNTs, and SWTlNNTs, respectively. It can be assumed that Equation (20) leads to an accurate evaluation of the surface shear modulus of the 13th group atom-nitride nanotubes across the entire range of the NT diameters considered in the current study, although the error obtained for NTs with $D_{n}$ up to 1.5 nm is slightly higher. The $G_{S}$ values calculated by Equation (20) are shown in Figure 9b as a function of the bond length, $a_{A13-N}$.

Similar to what was established for the surface Young’s modulus, the surface shear modulus $G_{S}$ values decrease gradually with increasing bond length after an initial drop when moving from SWBNNTs to SWAlNNTs.

**Figure 10:**
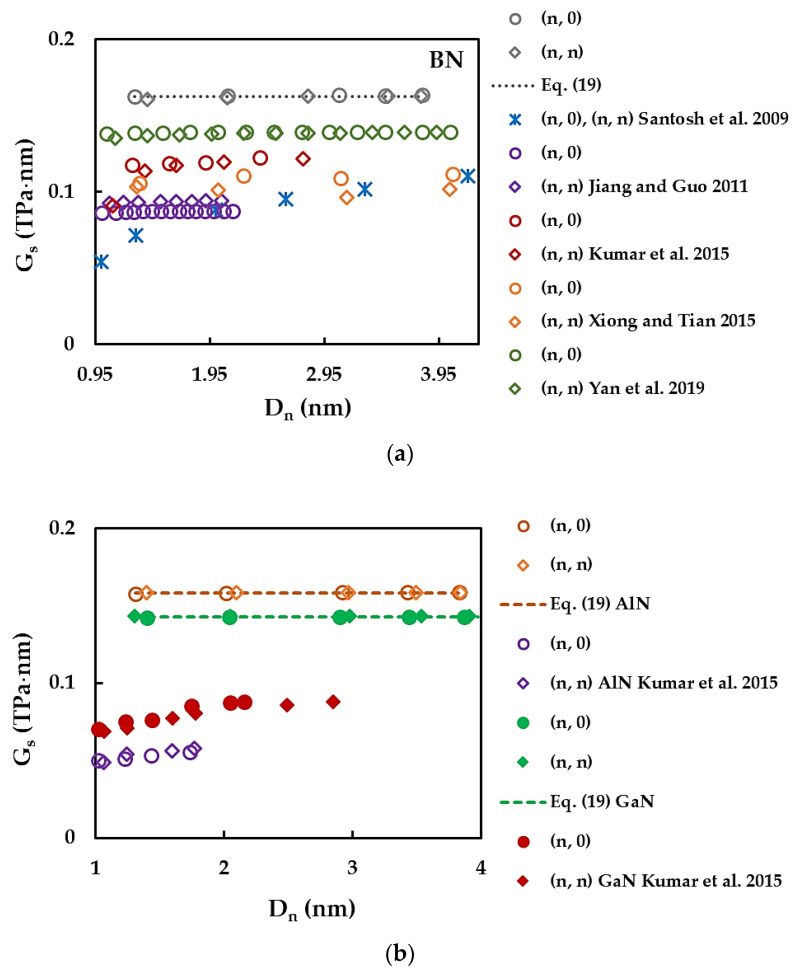
Comparison of the current surface shear modulu, $G_{S}$ evolutions with those available in the literature for (**a**) SWBNNTs and (**b**) SWAlNNTs and SWGaNNTs as a function of the NT diameter, $D_{n}$ [52,54,57,61,71].

The $G_{S}$ results available in the literature are scarce, even for the case of SWBNNTs, and show considerable discrepancy, as seen in Figure 10a,b. Xiong and Tian [54], Kumar et al. [52], Santosh et al. [57], and Yan et al. [61] reported shear modulus G values. To enable a comparison, the respective surface shear modulus was calculated by using $G_{S}=Gt_{n}$ for an NT wall thickness of $t_{n}$ = 0.330 nm [52], 0.333 nm [61], and 0.340 nm [57]. Xiong and Tian [54] did not report any $t_{n}$ value, so in this case, $t_{n}$ = 0.34 nm [48] was used.

The Poisson’s ratio, ν, of nitride nanotubes was assessed by Equation (13) using the EI and GJ rigidities acquired from bending and torsional tests, respectively, and the $\beta_{A13-N}$ and $\gamma_{A13-N}$ fitting parameters in Table 5. A combination of this equation with expression (17) and (18) for the EI and GJ rigidities allows us to calculate ν as follows:

(21) $$\nu \;=\frac{\beta_{A13-N}}{\gamma_{A13-N}}\;-\;1,$$

whose expression is independent of the NT diameter.

**Figure 11:**
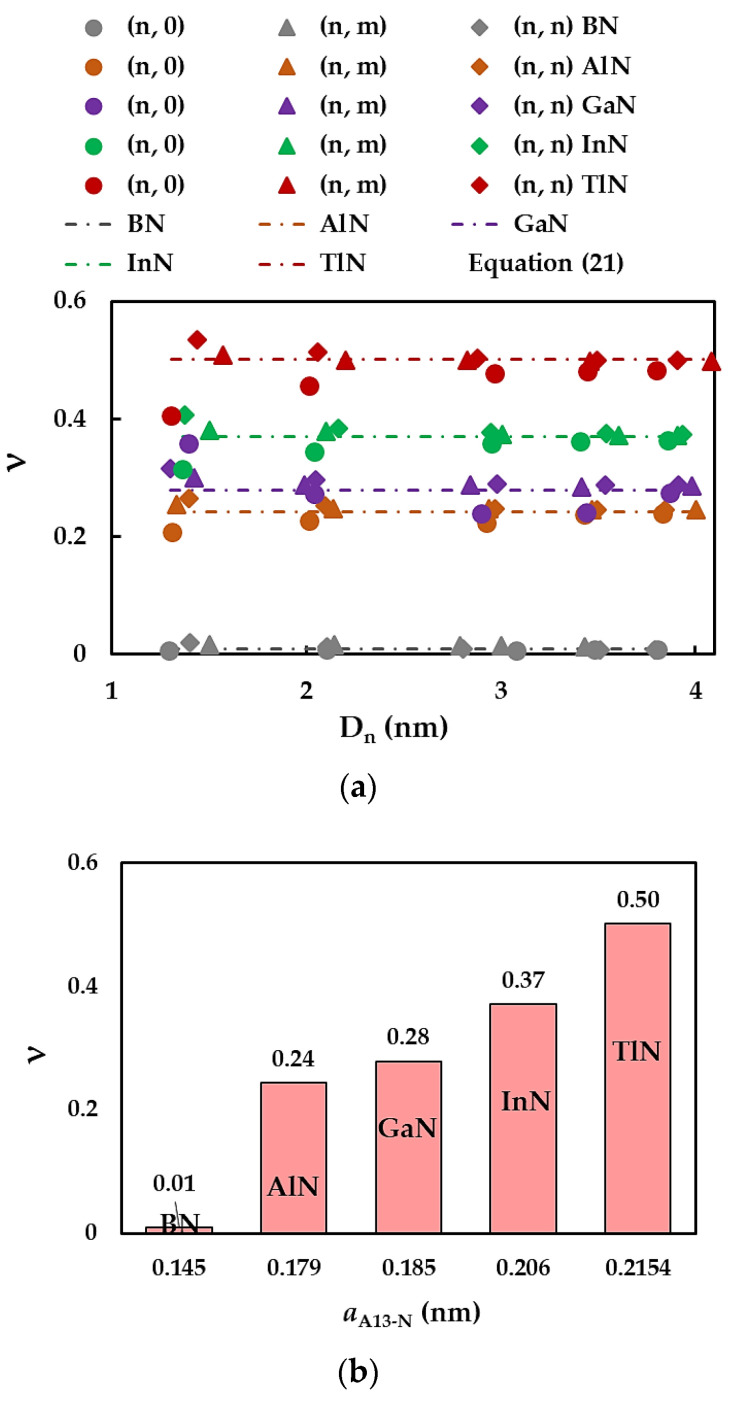
Evolutions of the Poisson’s ratio, ν, for SWBNNTs, SWAlNNTs, SWGaNNTs, SWInNNTs, and SWTlNNTs as a function of the (**a**) NT diameter, $D_{n}$, and (**b**) bond length, $a_{A13-N}$.

Figure 11a demonstrates the evolution of the Poisson’s ratio, ν, assessed by Equation (13), with the NT diameter, $D_{n}$, for the SWBNNTs, SWAlNNTs, SWGaNNTs, SWInNNTs, and SWTlNNTs in Table 3. The values of ν calculated by Equation (21) are also presented in Figure 11a. For zigzag, chiral, and armchair nitride NTs with a high value of $D_{n}$, ν converges to the constant value obtained using Equation (21). The higher the value of the bond length $a_{A13-N}$, the bigger the nanotube diameter $D_{n}^{\mathrm{st}}$ for which ν becomes stable (see Figure 11a). The $D_{n}^{\mathrm{st}}$ values are approximately 1.4 nm, 2.0 nm, 2.1 nm, 3.0 nm, and 3,4 nm for SWBNNTs, SWAlNNTs, SWGaNNTs, SWInNNTs, and SWTlNNTs, respectively. Similar qualitative results were reported for 13th group element-phosphide nanotubes [81]. For nitride NTs with diameters of $D_{n}$ < $D_{n}^{\mathrm{st}}$, the Poisson’s ratio increases for (n, 0) zigzag NTs, almost does not change for (n, m) chiral NTs, and decreases for (n, n) armchair NTs.

To understand the effect of first element (B, Al, Ga, In, Tl) of nitride NTs on the Poisson’s ratio, the values of ν assessed by Equation (21) are shown in Figure 11b as a function of the bond length, $a_{A13-N}$. The lowest value of ν = 0.01 was found for SWBNNTs. The Poisson’s ratio grows up to 0.24 when moving from SWBNNTs to SWAlNNTs; then, ν continues to increase with increasing bond length. The highest ν value equal to 0.50 is observed for the SWTlNNTs, whose value is about 50 times greater than that of SWBNNTs. This difference can be justified by the ratio between bending and torsional rigidities, EI/GJ, necessary to determine ν by Equation (13) and (21). In fact, the $\beta_{A13-N}$/$\gamma_{A13-N}$ ratio is nearly equal to 1 and 1.5 for the SWBNNTs and SWTlNNTs, respectively. This means that bending EI and torsional GJ rigidities are almost the same for the boron nitride NTs and EI > GJ for the thallium nitride NTs, resulting in a substantial increase in the ν value of SWTlNNTs. The increase in Poisson’s ratio with increasing bond length was reported in the studies by Jiang and Guo [63] for nitride and phosphide NTs and by Sakharova et al. [81] for phosphide NTs.

**Figure 12:**
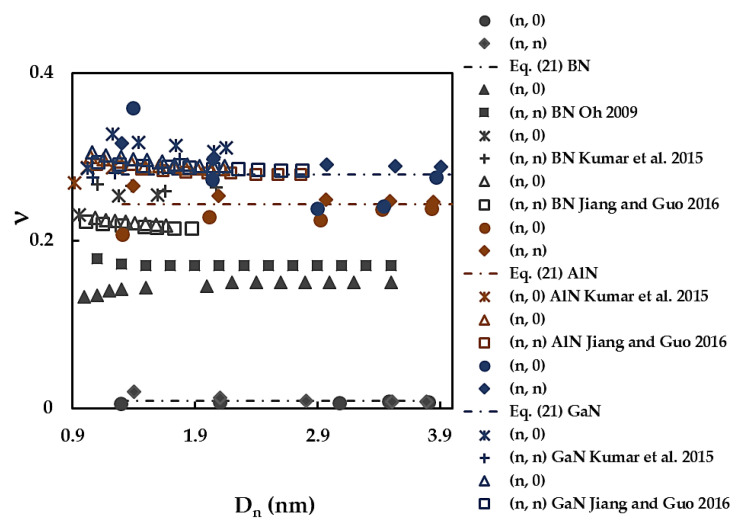
Comparison of the current evolutions of the Poisson’s ratio, ν, for SWBNNTs, SWAlNNTs, and SWGaNNTs with those reported by the other authors as a function of the NT diameter, $D_{n}$ [52,59,63].

Figure 12 compares the current Poisson’s ratio values with those available in the literature for the cases of SWBNNTs, SWAlNNTs, and SWGaNNTs. A good concordance, with a difference of ≈2%, is found when the ν value calculated by Equation (21) for SWGaNNTs is compared with that reported by Jiang and Guo [63] for the non-chiral GaN nanotubes. The Poisson’s ratios evaluated by Kumar et al. [52] for (n, 0) and (n, n) GaNNTs are ≈8% lower and ≈6% higher, respectively, than the ν values currently obtained. In other cases presented in Figure 12, there is a considerable scattering of the ν values.

Jiang and Guo [63] reported for both armchair and zigzag SWBNNTs, SWAlNNTs, and SWGaNNTs the trends in the Poisson’s ratio evolutions as a function of NTs diameter, $D_{n}$, for which ν decreases when $D_{n}$ increases; afterwards, the value of ν converges to an almost constant value (see Figure 12). This trend is in line with the present one for the evolutions of ν as a function of $D_{n}$, for armchair BN, AlN, and GaN nanotubes, although the decreasing rate found by Jiang and Guo [63] is slower.

## 4. Conclusions

The elastic properties, including the three rigidities, tensile, bending, and torsional; the surface Young’s and shear moduli; and the Poisson’s ratio of SWBNNTs, SWAlNNTs, SWGaNNTs, SWInNNTs, and SWTlNNTs, were evaluated in a numerical simulation study based on the NCM/MSM approach. The principal conclusions are specified below.

Analytical expressions, which allow for the evaluation of the three rigidities as a function of the NTs diameter and the fitting parameters without resorting to numerical simulation, were obtained for the most complete set of the 13th group atom-nitride nanotubes.

Also, the knowledge of these fitting parameters permits an accurate analytical assessment of the surface Young’s and shear modulus of the SWBNNTs, SWAlNNTs, SWGaNNTs, SWInNNTs, and SWTlNNTs with diameters $D_{n}$ of higher than 1.25 nm and the Poisson’s ratio, limiting the assessment to nanotubes with diameters $D_{n}$ > $D_{n}^{\mathrm{st}}$. The longer the bond length, the higher the value of $D_{n}^{\mathrm{st}}$, for which the Poisson’s ratio does not change with the increase in NT diameter.

The tensile, bending, and torsional rigidities, the surface Young’s and shear moduli, and the Poisson’s ratio of SWBNNTs, SWAlNNTs, SWGaNNTs, SWInNNTs, and SWTlNNTs are sensitive to the interatomic bond length of the hexagonal lattice. The three rigidities and the surface Young’s and shear moduli decrease, while the Poisson’s ratio increases with increasing bond length.

The results presented constitute a considerable contribution to references about the determination of the elastic properties of nitride nanotubes by analytical and numerical methods.

## Figures and Tables

**Table 1:** Bond length values of the 13th group element-nitride nanostructures available in the literature.

	BN	AlN	GaN	InN	TlN
$a_{A13-N}$, nm	0.1446 [52] 0.1447 [69] 0.145 [68] 0.147 [49] 0.151 [70] 0.153 [71]	0.177 [49] 0.179 [68] 0.1805 [1] 0.185 [72] 0.1856 [52] 0.193 [73] 0.195 [74]	0.175 [34] 0.184 [49] 0.185 [68] 0.1852 [1] 0.186 [72] 0.1863 [52] 0.194 [73]	0.203 [43] 0.206 [68] 0.2074 [1]	0.2154 [1] 0.224 [45] 0.230 [4]

**Table 2:** Chiral indices (n, m) and diameters, $D_{n}$, of the SWBNNTs, SWAlNNTs, SWGaNNTs, SWInNNTs, and SWTlNNTs.

NT Type	SWBNNTs		SWAlNNTs		SWGaNNTs		SWInNNTs		SWTlNNTs	
	(n, m)	D_n_, nm *	(n, m)	D_n_, nm *	(n, m)	D_n_, nm *	(n, m)	D_n_, nm	(n, m)	D_n_, nm
zigzag, θ = 0°	(16, 0)	1.297	(13, 0)	1.312	(13, 0)	1.398	(12, 0)	1.363	(11, 0)	1.306
	(26, 0)	2.107	(20, 0)	2.018	(19, 0)	2.043	(18, 0)	2.044	(17, 0)	2.019
	(38, 0)	3.080	(29, 0)	2.926	(27, 0)	2.903	(26, 0)	2.953	(25, 0)	2.969
	(43, 0)	3.485	(34, 0)	3.430	(32, 0)	3.440	(30, 0)	3.407	(29, 0)	3.444
	(47, 0)	3.809	(38, 0)	3.834	(36, 0)	3.870	(34, 0)	3.862	(32, 0)	3.800
chiral, θ = 19.1°	(14, 7)	1.501	(10, 5)	1.335	(10, 5)	1.422	(10, 5)	1.502	(10, 5)	1.571
	(20, 10)	2.144	(16, 8)	2.136	(14, 7)	1.991	(14, 7)	2.103	(14, 7)	2.199
	(26, 13)	2.788	(22, 11)	2.936	(20, 10)	2.844	(20, 10)	3.005	(18, 9)	2.828
	(28, 14)	3.002	(26, 13)	3.470	(24, 12)	3.413	(24, 12)	3.606	(22, 11)	3.456
	(32, 16)	3.431	(30, 15)	4.004	(28, 14)	3.982	(26, 13)	3.906	(26, 13)	4.085
armchair, θ = 30°	(10, 10)	1.404	(8, 8)	1.398	(7, 7)	1.303	(7, 7)	1.377	(7, 7)	1.440
	(15, 15)	2.106	(12, 12)	2.097	(11, 11)	2.048	(11, 11)	2.164	(10, 10)	2.057
	(20, 20)	2.807	(17, 17)	2.971	(16, 16)	2.979	(15, 15)	2.951	(14, 14)	2.880
	(25, 25)	3.509	(20, 20)	3.495	(19, 19)	3.538	(18, 18)	3.541	(17, 17)	3.497
	(27, 27)	3.790	(22, 22)	3.845	(21, 21)	3.910	(20, 20)	3.934	(19, 19)	3.908

* The diameters, $D_{n}$, of SWBNNTs, SWAlNNTs, and SWGaNNTs are calculated adopting the bond lengths $a_{B-N}$ = 0.147 nm, $a_{\mathrm{Al}-N}$ = 0.183 nm, and $a_{\mathrm{Ga}-N}$ = 0.195 nm, respectively, as defined by Nanotube Modeler© software (version 1.8.0, ©JCrystalSoft, http://www.jcrystal.com, 1 March 2024); for the SWInNNTs and SWTlNNTs, the bond lengths $a_{\mathrm{In}-N}$ = 0.206 nm [68] and $a_{\mathrm{Tl}-N}$ = 0.2154 nm [1] were assumed.

**Table 3:** Bond length, surface Young’s modulus, and Poisson’s ratio and $k_{r}$, $k_{\theta }$, and $k_{\tau }$ force field constants for BN, AlN, GaN, InN, and TlN nanotubes.

Compound	$a_{A13-N}$, nm [68]	E_s_*,* nN/nm [68]	ν [68]	$k_{r}$, nN/nm	$k_{\theta }$, nN·nm/rad^2^	$k_{\tau }$, nN·nm/rad^2^
BN	0.145	267	0.21	585	0.994	2.470
AlN	0.179	116	0.46	372	0.451	0.625
GaN	0.185	110	0.48	366	0.445	
InN	0.206	67	0.59	283	0.296	
TlN	0.2154 *	34.5 *	0.689 *	192	0.151	

* Values from Ye and Peng [1].

**Table 4:** Geometrical and elastic properties of beams as input parameters in FE simulations.

Compound	Diameter, d, nm	Formulation	Young’s Modulus, E_b_, GPa	Formulation	Shear Modulus, G_b_, GPa	Formulation	$\mathrm{Poisson}’s\;\mathrm{Ratio},\;\nu_{b\;}$
BN	0.1648	$d=4\sqrt{\frac{k_{\theta }}{k_{r}}}$	3977	$E_{b}=\frac{k_{r}^{2}l}{4\pi k_{\theta }}$	4941	$G_{b}=\frac{{k_{r}^{2}k}_{\tau }l}{8\pi k_{\theta }^{2}}$	0.21 [68]
AlN	0.1392		4374		3032		0.46 [68]
GaN	0.1395		4437		3113		0.48 [68]
InN	0.1294		4432		4674		0.59 [68]
TlN	0.1120		4200		8712		0.689 [1]

**Table 5:** Fitting parameters $\alpha_{A13-N}$, $\beta_{A13-N}$, and $\gamma_{A13-N}$ for SWBNNTs, SWAlNNTs, SWGaNNTs, SWInNNTs, and SWTlNNTs; mean difference between the EA, EI, and GJ rigidities calculated resourcing to these parameters (Equations (16)–(18)) and the respective rigidities obtained from FEA.

Compound	Fitting Parameters			Mean Difference, %		
	$\alpha_{A13-N}$, nN/nm	$\beta_{A13-N}$, nN/nm	$\gamma_{A13-N}$, nN/nm	EA, nN	EI, nN·nm^2^	GJ, nN·nm^2^
BN	1029.96 ^1^	128.08 ^1^	126.93 ^1^	0.08	0.23	0.08
AlN	497.38	62.09	49.94	0.23	0.36	0.31
GaN	448.92	55.99	43.78	0.24	0.34	0.58
InN	321.72 ^1^	40.14 ^1^	29.28 ^1^	0.32	0.49	0.31
TlN	175.71	21.92	14.60	0.41	0.56	0.54

^1^ The values of the fitting parameters for the BNNTs and InNNTs are similar to those obtained in the author’s previous works [48,60].

**Table 6:** Comparison of the current Young’s modulus results with the experimental values available in the literature.

Reference	Method	Type of NTs	E, TPa			Comments
			Reference	Current	Difference, %	
Arenal et al. [64]	HRTEM-AFM+ analytical	SWBNNTs	1.11 ± 0.17	0.968 ^1^	12.8	t_n_ = 0.07 nm
Tanur et al. [65]	AFM: a three-point bending + analytical	MWBNNTs	0.760 ± 0.03		21.4	outer diameter in the range of 18 to 55 nm
Zhou et al. [66]	HRTEM	MWBNNTs	0.906		6.8	outer diameter in the range of 28 to 57 nm
Chen et al. [67]	TEM + analytical	MWBNNTs	1.050		7.8	outer diameter of 37.34 nm and 40 layers
Stan et al. [32]	CR-AFM + FE analysis	faceted AlNNTs with triangular cross-section	0.3252 ± 0.015	0.385 ^1^	18.5	inner facet
Hung et al. [38]	nanoindentation + analytical	SWGaNNTs	0.484	0.418 ^1^	13.6	NT length of 500 nm

^1^ Calculated from the surface Young’s modulus, $E_{S}$, using the equality $E=E_{S}/t_{n}$ for an SWBNNT wall thickness of $t_{n}$ = 0.340 nm [48]; an SWAlNNT wall thickness of $t_{n}$ = 0.410 nm [50]; and for an SWGaNNT wall thickness of $t_{n}$ = 0.342 nm [84].

## Data Availability

The data presented in this study are available upon request from the corresponding author after obtaining permission from the authorised person.

## References

[1] Ye C., Peng Q. (2023). Mechanical Stabilities and Properties of Graphene-like 2D III-Nitrides: A Review. Crystals.

[2] Zheng F., Xiao X., Xie J., Zhou L., Li Y., Dong H. (2022). Structures, properties and applications of two-dimensional metal nitrides: From nitride MXene to other metal nitrides. 2D Mater..

[3] Abdullah N.R., Abdullah B.J., Gudmundsson V. (2022). Electronic and optical properties of metallic nitride: A comparative study between the MN (M = Al, Ga, In, Tl) monolayers. Solid State Commun..

[4] Elahi S.M., Farzan M., Salehi H., Abolhasani M.R. (2016). An investigation of electronic and optical properties of TlN nanosheet and compare with TlN bulk (Wurtzite) by first principle. Optik.

[5] Golberg D., Bando Y., Huang Y., Terao T., Mitome M., Tang C., Zhi C. (2010). Boron nitride nanotubes and nanosheets. ACS Nano.

[6] Wang Y., Zhou V., Xie Y., Zheng X.-Q., Feng P.X.-L. (2019). Optical contrast signatures of hexagonal boron nitride on a device platform. Opt. Mater. Express..

[7] Song L., Ci L., Lu H., Sorokin P.B., Jin C., Ni J., Kvashnin A.G., Kvashnin D.G., Lou J., Yakobson B.I. (2010). Large scale growth and characterization of atomic hexagonal boron nitride layers. Nano Lett..

[8] Vurgaftman I., Meyer J.R. (2003). Band parameters for nitrogen-containing semiconductors. J. Appl. Phys..

[9] Chattopadhyay S., Ganguly A., Chen K.-H., Chen L.-C. (2009). One-Dimensional Group III-Nitrides: Growth, Properties, and Applications in Nanosensing and Nano-Optoelectronics. Crit. Rev. Solid State Mater. Sci..

[10] Zaoui A. (2003). Plane wave pseudopotential study of ground state properties and electrochemical description of thallium nitride. Mater. Sci. Eng. B.

[11] Li-Wei S., Yi-Feng D., Li-Xia Q. (2010). Structural Stability and Elastic Properties of Wurtzite TlN under Hydrostatic Pressure. Chin. Phys. Lett..

[12] Walker K.E., Rance G.A., Pekker A., Tóháti H.M., Fay M.W., Lodge R.W., Stoppiello C.T., Kamarás K., Khlobystov A.N. (2017). Growth of carbon nanotubes inside boron nitride nanotubes by coalescence of fullerenes: Toward the world’s smallest coaxial cable. Small Methods.

[13] Huang Z., Lü T.-Y., Wang H.-Q., Yang S.-W., Zheng J.-C. (2017). Electronic and thermoelectric properties of the group-III nitrides (BN, AlN and GaN) atomic sheets under biaxial strains. Comput. Mater. Sci..

[14] Amorim B., Cortijo A., de Juan F., Grushin A.G., Guinea F., Gutiérrez-Rubio A., Ochoa H., Parente V., Roldán R., San-José P. (2016). Novel effects of strains in graphene and other two dimensional materials. Phys. Rep..

[15] Behzad S. (2017). Effects of strain and thickness on the electronic and optical behaviors of two-dimensional hexagonal gallium nitride. Superlattices Microstruct..

[16] Liu P., Sarkar A.D., Ahuja R. (2014). Shear strain induced indirect to direct transition in band gap in AlN monolayer nanosheet. Comput. Mater. Sci..

[17] Beshkova M., Yakimova R. (2020). Properties and potential applications of two-dimensional AlN. Vacuum.

[18] Chowdhury R., Adhikari S. (2011). Boron-nitride nanotubes as zeptogram-scale bionanosensors: Theoretical investigations. IEEE Trans. Nanotechnol..

[19] Noei M., Soleymanabadi H., Peyghan A.A. (2017). Aluminum nitride nanotubes. Chem. Pap..

[20] Albarakati R., Al-Qurashi O., Safi Z., Wazzan N. (2023). A dispersion-corrected DFT calculation on encapsulation of favipiravir drug used as antiviral against COVID-19 into carbon-, boron-, and aluminum-nitride nanotubes for optimal drug delivery systems combined with molecular docking simulations. Struct Chem..

[21] Liu B., Bando Y., Wang M., Tang C., Mitome M., Golberg D. (2009). Crystallography and elasticity of individual GaN nanotubes. Nanotechnology.

[22] Rubio A., Corkill J., Cohen M.L. (1994). Theory of graphitic boron nitride nanotubes. Phys. Rev. B.

[23] Lourie O.R., Jones C.R., Bartlett B.M., Gibbons P.C., Ruoff R.S., Buhro W.E. (2000). CVD growth of boron nitride nanotubes. Chem. Mater..

[24] Ahmad P., Khandaker M.U., Khana Z.R., Amina Y.M. (2015). Synthesis of boron nitride nanotubes via chemical vapour deposition: A comprehensive review. RSC Adv..

[25] Kim J., Lee S., Uhm Y.R., Jun J., Rhee C.K., Kim G.M. (2011). Synthesis and growth of boron nitride nanotubes by a ball milling–annealing process. Acta Mater..

[26] Golberg D., Bando Y., Eremets M., Takemura K., Kurashima K., Yusa H. (1996). Nanotubes in boron nitride laser heated at high pressure. Appl. Phys. Lett..

[27] Kim K.S., Couillard M., Shin H., Plunkett M., Ruth D., Kingston C.T., Simard B. (2018). Role of hydrogen in high-yield growth of boron nitride nanotubes at atmospheric pressure by induction thermal plasma. ACS Nano.

[28] Zhang D., Zhang R. (2003). Theoretical prediction on aluminum nitride nanotubes. Chem. Phys. Lett..

[29] Wu Q., Hu Z., Wang X., Lu Y., Chen X., Xu H., Chen Y. (2003). Synthesis and characterization of faceted hexagonal aluminum nitride nanotubes. J. Am. Chem. Soc..

[30] Balasubramanian C., Bellucci S., Castrucci P., De Crescenzi M., Bhoraskar S. (2004). Scanning tunneling microscopy observation of coiled aluminum nitride nanotubes. Chem. Phys. Lett..

[31] Yin L.W., Bando Y., Zhu Y.C., Li M.S., Tang C.-C., Golberg D. (2005). Single-crystalline AlN nanotubes with carbon-layer coatings on the outer and inner surfaces via a multiwalled-carbon-nanotubetemplate-induced route. Adv. Mater..

[32] Stan G., Ciobanu C.V., Thayer T.P., Wang G.T., Creighton J.R., Purushotham K.P., Bendersky L.A., Cook R.F. (2009). Elastic moduli of faceted aluminum nitride nanotubes measured by contact resonance atomic force microscopy. Nanotechnology.

[33] Fan Y. (2011). Formation of crystalline AlN nanotubes by a roll-up approach. Mater. Lett..

[34] Lee S.M., Lee Y.H., Hwang Y.G., Elsner J., Porezag D., Frauenheim T. (1999). Stability and electronic structure of GaN nanotubes from density-functional calculations. Phys. Rev. B.

[35] Goldberger J., He R., Zhang Y., Lee S., Yan H., Choi H.-J., Peidong Y. (2003). Single-crystal gallium nitride nanotubes. Nature.

[36] Yin L.W., Bando Y., Zhu Y.C., Golberg D., Yin L.W., Li M.S. (2004). Indium-assisted synthesis on GaN nanotubes. Appl. Phys. Lett..

[37] Hu J.Q., Bando Y., Golberg D., Liu Q.L. (2003). Gallium Nitride Nanotubes by the Conversion of Gallium Oxide Nanotubes. Angew. Chem. Int. Ed..

[38] Hung S.-C., Su Y.-K., Fang T.-H., Chang S.-J., Ji L.-W. (2005). Buckling instabilities in GaN nanotubes under uniaxial compression. Nanotechnology.

[39] Liu B.D., Bando Y., Tang C.C., Shen G.Z., Golberg D., Xu F.F. (2006). Wurtzite-type faceted single-crystalline GaN nanotubes. Appl. Phys. Lett..

[40] Jung W.-G., Jung S.-H., Kung P., Razeghi M. (2006). Fabrication of GaN nanotubular material using MOCVD with an aluminium oxide membrane. Nanotechnology.

[41] Yin L., Bando Y., Golberg D., Li M. (2004). Growth of single-crystal indium nitride nanotubes and nanowires by controlled-carbonitridation reaction route. Adv. Mater..

[42] Sardar K., Deepak F.L., Govindaraj A., Seikh M.M., Rao C.N.R. (2005). InN nanocrystals, nanowires, and nanotubes. Small.

[43] Qian Z., Hou S., Zhang J., Li R., Shen Z., Zhao X., Xue Z. (2005). Stability and electronic structure of single-walled InN nanotubes. Phys. E.

[44] Shah E.V., Roy D.R. (2019). Density functional investigation on hexagonal nanosheets and bulk thallium nitrides for possible thermoelectric applications. Appl. Nanosci..

[45] Li X., Dai Y., Ma Y., Wei W., Yu L., Huang B. (2015). Prediction of large-gap quantum spin hall insulator and Rashba-Dresselhaus effect in two-dimensional g-TlA (A = N, P, As, and Sb) monolayer films. Nano Res..

[46] Peng Q., Liang C., Ji W., De S. (2012). A First Principles Investigation of the Mechanical Properties of *g*-TlN. Model. Numer. Simul. Mater. Sci. (MNSMS).

[47] Antunes J.M., Pereira A.F.G., Sakharova N.A. (2022). Overview on the Evaluation of the Elastic Properties of Non-Carbon Nanotubes by Theoretical Approaches. Materials.

[48] Sakharova N.A., Antunes J.M., Pereira A.F.G., Chaparro B.M., Fernandes J.V. (2021). On the determination of elastic properties of single-walled boron nitride nanotubes by numerical simulation. Materials.

[49] Kochaev A. (2017). Elastic properties of noncarbon nanotubes as compared to carbon nanotubes. Phys. Rev. B.

[50] Hao J.-H., Wang Y.-F., Yin Y.-H., Jiang R., Wang Y.-F., Jin Q.-H. (2015). An ab initio study of the size-dependent mechanical behavior of single-walled AlN nanotubes. Solid State Sci..

[51] Fabris G.S.L., Paskocimas C.A., Sambrano J.R., Paupitz R. (2021). One- and two-dimensional structures based on gallium nitride. J. Solid State Chem..

[52] Kumar D., Verma V., Dharamvir K., Bhatti H.S. (2015). Elastic moduli of boron nitride, aluminium nitride and gallium nitride nanotubes using second generation reactive empirical bond order potential. Multidiscip. Model. Mater. Struct..

[53] Jeng Y.-R., Tsai P.C., Fang T.-H. (2004). Molecular dynamics investigation of the mechanical properties of gallium nitride nanotubes under tension and fatigue. Nanotechnology.

[54] Xiong Q.-l., Tian X.G. (2015). Torsional properties of hexagonal boron nitride nanotubes, carbon nanotubes and their hybrid structures: A molecular dynamics study. AIP Adv..

[55] Tao J., Xu G., Sun Y. (2015). Elastic properties of boron-nitride nanotubes through an atomic simulation method. Math. Prob. Eng..

[56] Xu B., Lu J.A., Pan B.C., Yu Q.X. (2005). Atomic structures and mechanical properties of single-crystal GaN nanotubes. Phys. Rev. B.

[57] Santosh M., Maiti P.K., Sood A.K. (2009). Elastic properties of boron nitride nanotubes and their comparison with carbon nanotubes. J. Nanosci. Nanotech..

[58] Le M.-Q. (2014). Young’s modulus prediction of hexagonal nanosheets and nanotubes based on dimensional analysis and atomistic simulations. Meccanica.

[59] Oh E.-S. (2010). Elastic properties of boron-nitride nanotubes through the continuum lattice approach. Mater. Lett..

[60] Sakharova N.A., Pereira A.F.G., Antunes J.M., Chaparro B.M., Fernandes J.V. (2023). On the determination of elastic properties of indium nitride nanosheets and nanotubes by numerical simulation. Metals.

[61] Yan J.W., He J.B., Tong L.H. (2019). Longitudinal and torsional vibration characteristics of boron nitride nanotubes. J. Vib. Eng. Technol..

[62] Genoese A., Genoese A., Salerno G. (2019). On the nanoscale behaviour of single-wall C, BN and SiC nanotubes. Acta Mech..

[63] Jiang L., Guo W. (2016). Analytical solutions for elastic binary nanotubes of arbitrary chirality. Acta Mech. Sin..

[64] Arenal R., Wang M.-S., Xu Z., Loiseau A., Golberg D. (2011). Young modulus, mechanical and electrical properties of isolated individual and bundled single-walled boron nitride nanotubes. Nanotechnology.

[65] Tanur A.E., Wang J., Reddy A.L.M., Lamont D.N., Yap Y.K., Walker G.C. (2013). Diameter-dependent bending modulus of individual multiwall boron nitride nanotubes. J. Phys. Chem. B.

[66] Zhou X., Tang D.-M., Mitome M., Bando Y., Sasaki T., Golberg D. (2019). Intrinsic and defect-related elastic moduli of boron nitride nanotubes as revealed by in situ Transmission Electron Microscopy. Nano Lett..

[67] Chen G., Lu H., Cui J., Yu H., Wang B., Liu Y., Li H., Jiang N. (2019). In situ real-time study buckling behavior of boron nitride nanotubes with axial compression by TEM. Chin. Chem. Lett..

[68] Şahin H., Cahangirov S., Topsakal M., Bekaroglu E., Akturk E., Senger R.T., Ciraci S. (2009). Monolayer honeycomb structures of group-IV elements and III-V binary compounds: First-principles calculations. Phys. Rev. B.

[69] Tapia A., Cab C., Hernández-Pérez A., Villanueva C., Peñuñuri F., Avilés F. (2017). The bond force constants and elastic properties of boron nitride nanosheets and nanoribbons using a hierarchical modeling approach. Phys. E.

[70] Menon M., Srivastava D. (1999). Structure of boron nitride nanotubes: Tube closing versus chirality. Chem. Phys. Lett..

[71] Jiang L., Guo W. (2011). A molecular mechanics study on size-dependent elastic properties of single-walled boron nitride nanotubes. J. Mech. Phys. Solids.

[72] Kang J.W., Hwang H.J. (2004). Atomistic study of III-nitride nanotubes. Comput. Mater. Sci..

[73] Huber K.P., Hertzberg G. (1979). Molecular Spectra and Molecular Siructure: IV. Constants of Diatomic Molecules.

[74] Zhou Z., Zhao J., Chen Y., Schleyer P.R., Chen Z. (2007). Energetics and electronic structures of AlN nanotubes/wires and their potential application as ammonia sensors. Nanotechnology.

[75] Li C., Chou T.W. (2003). A structural mechanics approach for the analysis of carbon nanotubes. Int. J. Solids Struct..

[76] Genoese A., Genoese A., Rizzi N.L., Salerno G. (2018). Force constants of BN, SiC, AlN and GaN sheets through discrete homogenization. Meccanica.

[77] Ansari R., Rouhi S., Mirnezhad M., Aryayi M. (2015). Stability characteristics of single-walled boron nitride nanotubes. Arch. Civ. Mech. Eng..

[78] Mayo S.L., Barry D., Olafson B.D., Goddard W.A. (1990). DREIDING: A generic force field for molecular simulations. J. Phys. Chem..

[79] Sakharova N.A., Pereira A.F.G., Antunes J.M., Brett C.M.A., Fernandes J.V. (2015). Mechanical characterization of single-walled carbon nanotubes. Numerical simulation study. Compos. B-Eng..

[80] Pereira A.F.G., Antunes J.M., Fernandes J.V., Sakharova N.A. (2016). Shear modulus and Poisson’s ratio of single-walled carbon nanotubes: Numerical evaluation. Phys. Status Solidi B.

[81] Sakharova N.A., Antunes J.M., Pereira A.F.G., Chaparro B.M., Fernandes J.V. (2022). Elastic properties of single-walled phosphide nanotubes: Numerical Simulation Study. Nanomaterials.

[82] Sakharova N.A., Pereira A.F.G., Antunes J.M. (2022). Elastic moduli of non-chiral singe-walled silicon carbide nanotubes: Numerical simulation study. Materials.

[83] Fernandes J.V., Pereira A.F.G., Antunes J.M., Chaparro B.M., Sakharova N.A. (2023). Numerical Simulation Study of the Mechanical Behaviour of 1D and 2D Germanium Carbide and Tin Carbide Nanostructures. Materials.

[84] Hess P. (2020). Thickness of elemental and binary single atomic monolayers. Nanoscale Horiz..

